# Culture and mental health resilience in times of COVID-19

**DOI:** 10.1007/s00148-021-00840-7

**Published:** 2021-05-19

**Authors:** Annie Tubadji

**Affiliations:** Swansea University, Bay Campus, Swansea, SA1 8EN UK

**Keywords:** Culture, Mental health, Anxiety, Happiness, Social capital, COVID-19, Z10, R11, H51, I18, N30, P36

## Abstract

This paper aims to clarify the role of culture as a public good that serves to preserve mental health. It tests the evolutionary hypothesis that cultural consumption triggers a microeconomic mechanism for the self-defense of mental health from uncertainty. The COVID-19 pandemic offers a natural experiment of cultural consumption under increased uncertainty. Using primary data from a pilot survey conducted online during the pandemic and applying Probit and Heckman selection models, the study analyzes levels of happiness and propensity to help others. The results suggest that past consumption of culture is associated with higher happiness levels during crises. Moreover, spontaneous cultural practices (such as group singing) during times of uncertainty are associated with an increase in the pro-social propensity to help others. These findings highlight culture as a tool for promoting mental health at the micro level and social capital resilience at the aggregate level.

## Introduction

During economic shocks, the cultural sector is generally left by policymakers to the mercy of serendipity, and, with few exceptions, it is traditionally perceived as a needy industry that is among the first candidates for austerity measures. The question raised by this paper is whether this attitude is economically justified, in particular whether the essential role of culture as a public good with implications for individual mental health is overlooked in economic research. To answer this question, the definition and function of culture needs to be re-evaluated, as the notion is still loosely understood in contemporary economics.

Definitionally, some studies address culture as rules, norms, and traditions (see, for example, Guiso et al. 2006; Alesina and Giuliano 2015). Others accentuate its behavioral side as goods and services produced and consumed in the process of cultural participation (see, for a leading example, Throsby 1999). Inspired by Scitovsky (1983, 1972, 1976), the culture-based development (CBD) definition of culture unites the former two perspectives by building on the intrinsic link between them. Namely, CBD defines culture as a “story of stories,” a complex entity that is used to justify certain rules of behavior. This entity is embodied in norms and beliefs and material goods and services. CBD also draws a temporal divide between past culture (of both material and immaterial kind), termed cultural heritage, and present norms and beliefs and their related goods and services, termed living culture (see Tubadji 2012, 2013, 2020a, b, c; Tubadji and Montalto 2020).^1^ This study adopts the CBD definition of culture.

Functionally, culture is usually addressed as a mixed good that is partially a luxury good (necessary only after basic needs are satisfied) (see Heilbrun and Gray 2001) and partially a public good (with benefits for all and endowing individuals with useful cultural capital) (see Bourdieu 1986). The latter helps in social mobility and optimizes the utilization of our social networking (Bourdieu 1973). In line with Denzau and North (2000), CBD recombines these two aspects and postulates that the main function of culture is to alleviate pain from uncertainty by creating predictable behavior according to present cultural norms and rules for all. The main channel for the implementation of this function is cultural consumption, which, in the spirit of Veblenian consumption (Veblen 1899), is a manner of signaling compliance with cultural norms and rules. Thus, according to CBD, the greater the cultural consumption by individuals and society, the lesser the depletion from the pain of uncertainty as part of mental health.^2^

Mental health is a spectrum of states, but being in a balanced position mentally is an essential need for individuals and society. This paper argues that the consumption of cultural goods and services (i.e., cultural participation) can serve as a tool for keeping the individual centered around the golden mean of mental health. According to Schelling’s model (1969, 1978) of individual and system behavior, cultural consumption is expected to affect the mental health of the entire population. However, while much is known about the various impacts of culture on the aggregate level,^3^ microeconomic mental health–related mechanisms behind these economic effects are still under-researched. This oversight may be harmful if it leads policymakers to neglect the public good role of culture.

The shock of COVID-19 represents a unique natural experiment about mental health and cultural consumption under increased uncertainty. The mental health of people across the world was significantly affected by the lockdown imposed due to COVID-19 (see Tubadji et al. 2020a, b, c, d, e, f; Yamamura and Tsustsui 2021). As the crisis struck and the lockdown was imposed, concert halls, museums, and even some of the Egyptian pyramids granted free online access for people. Moreover, funding for the cultural sector became a secondary issue at the policymaking level in most countries,^4^ with the exceptions of Germany and New Zealand. Thus, what is urgently needed is empirical evidence on whether public cultural expenditure is now indeed redundant for the economy or, instead, fully justified. This paper uses the natural experiment of COVID-19 to explore the effect of cultural consumption under uncertainty.

The current paper aims to tap into this natural experiment to study the CBD micromechanism. Namely, using primary data from a pilot online survey carried out March 23–29, 2020, the paper employs a hedonic modeling approach to address the existence of the cultural micromechanism of pain alleviation and support for mental health resilience during increased uncertainty. It relies on various measures of happiness (Kahneman and Krueger 2006; Frey and Stuetzer 2018; Weimann et al. 2015) as well as a measure of pro-social propensities in human behavior, treating them as alternative approximations of mental health. The paper addresses two aspects of the cultural impact on mental health resilience: the cultural effect on mental health prevention (i.e., the effects of past consumption of culture on the levels of happiness during the COVID-19 pandemic) and the cultural impact on the resilience of the community spirit (in line with Guiso et al. 2008, 2016) approximated by the change in the social capital propensity in human behavior due to cultural consumption during the lockdown period.

The paper finds strong evidence for an association between cultural consumption from pre-pandemic periods and individual levels of happiness during the pandemic. Additionally, during increased uncertainty, group cultural engagement is associated with a boost in people’s pro-social behavior. The “Results” section discusses potential macroeconomic implications.

The structure of the paper is as follows. Section 2 offers an overview from an evolutionary and behavioral economics perspective about the role of culture as an essential tool for alleviating pain from uncertainty. Section 3 outlines the neuroscience motivation for considering culture as a tool for supporting psychological resilience under shock conditions. Section 4 explains the CBD micromechanism that is part of the utility function of the general consumer, affecting happiness and mental health resilience. This section also outlines some regional and macroeconomic implications of this micro mechanism. Section 5 offers an empirical test of the CBD micromechanism. Section 6 concludes and reflects on some fiscal policy implications concerning culture.

## Evolutionary view on culture as part of the essentials

To understand the role of culture for mental health, one needs to look further into evolutionary evidence. The evolutionary perspective on culture here refers to the way that people intuitively have used culture in the socio-economic life over the centuries. On the one hand, culture is clearly a consumption good with major relation to people’s mental health. The theater of the oppressed is a known tool used for mental health support (Boal 1974). Painting, poetry, and music have been used as tools for mental recovery of recidivists and criminals in prisons (Gussak 2006; Johnson 2008). Painters are known to have been painting what they do not have in their lives, and even more broadly—neuroscience has documented that music can improve the happiness of an average healthy person within minutes (Redgrave 1878; Koen 2008; De Botton and Armstrong 2013). Finally, music has been part of the lives of the first people, which obviously points towards the role of culture among the essential needs, rather than among the luxury goods (Huron 2001; Grewe et al. 2009; Wallin et al. 2001; Bannan 2012; Guiso et al. 2016; Morley 2013; Patel 2006).

Meanwhile, over the centuries, the access to culture might have become a luxury for some members of society. Yet, the current study interprets this as just another aspect of the developing stark inequalities in redistribution over time. The lack of awareness among the general public and among policymakers about the importance of the inequality in cultural consumption only aggravates this type of inequality.^5^ This can be especially consequential in terms of the cultural capital endowment among the different socio-economic strata, which leads to sticky cultural tastes and sluggish social mobility (Bourdieu 1986; Georg 2004; Bennett and Silva 2006; van Hek and Kraaykamp 2013; Oakley and O’Brien 2015; Veal 2016; Gomes and Librero-Cano 2018; Katz-Gerro et al. 2009). Overseeing the role of access to cultural consumption for mental health can only add to this basket of inequalities in our modern world Abel (2008).

The above wealth of evidence on the role of culture in human behavior can be synthesized in the hypothesis that the effect of culture on mental health exists for one very important reason: the role that culture plays in alleviating the pain from uncertainty. As known from innovation and economic studies, and behavioral economics more generally, uncertainty is a major factor in human behavior (Kahneman and Tversky 1980). People are twice more strongly affected by the fear of loss, than by the greed for gain. A general tendency to avoid uncertainty also explains why a potential surprise function exists in human behavior, which prevents people from being sufficiently daring and innovative (Shackle 1949; Foldes 1958; Katzner 1986, 1989; Cantillo 2014; Derbyshire 2017). The feeling of uncertainty is itself very sensitive to cultural modification (see Tubadji et al. (2020d) for an extensive literature review). This is so because culture embodies a tailor-made set of socially affirmed immaterial beliefs and values, which do not have an intrinsic value but are socially constructed. Yet, boundedly, people prefer to believe in them as if they are intrinsic, in order for this set of rules to serve as a psychological tool for handling uncertainty (Delton et al. 2011; Tubadji 2020c). Namely, having a “certain” cultural compass of heuristics serves for an illusionary alleviation of our fear of the unknown and the lack of clear uncertainty avoidance strategy (Kahneman et al. 1991; Akerlof and Shiller 2010). Establishing a certain set of culturally tailored heuristics, institutionalized up to the rank of intrinsic social norms, beliefs, and attitudes, is a cognitive survival strategy for securing a mental health comfort zone for existence in an objectively uncertain world (Gudykunst 1995; Hirsh and Kang 2016). It is similar to the herd behavior in other mammals. According to Hall (1966), mammals generally tend to live in herds so that they signal danger to each other through one’s own certainly predictable behavior in the face of danger. Put differently, having established cultural rules makes people feel more certain what they have to do for their own good and what others will do in a culturally defined world. This makes them feel less uncertain about their environment where they try to survive. Moreover, evolutionarily, people have improved their smartness explicitly thanks to consuming culture (Boyd and Richerson 2005; Richerson and Boyd 2008; Henrich 2017). The current study focuses on the understanding that accumulating this culture-related mental comfort feeling in one’s psychological system over time, by consuming culture more intensely in normal periods, increases one’s resistance to mental depletion especially under shock conditions.

## Culture and psychological resilience under economic shocks

Psychological resilience is a concept very well known in psychological studies (Fletcher and Sarkar 2013). Behavioral economics has borrowed a lot from psychology; however, the notion of psychological resilience has not yet been sufficiently investigated in the contexts of economic thinking, while there are strong indications for its relevance (Graber et al. 2015).

Firstly, on the aggregate level, there are studies documenting the role of psychological types for local socio-economic development (Fritsch and Rusakova 2010; Obschonka et al. 2013; Stuetzer et al. 2014; Fritsch et al. 2019). Next, the topic of economic resilience is emerging and gaining higher speed and deeper understanding in regional economics (Martin 2012; Reggiani 2012; Modica and Reggiani 2015; O’Kelly 2015; Martin and Gardiner 2019; Nijkamp 2007; Murray 2020). While mental health is known to be subject to depletion (Zyphur et al. 2007; Ainsworth et al. 2014; Banker et al. 2017), the link between psychological (mental health) depletion during negative shocks in the economy (Zahran et al. 2011) and the aftermaths of mental resilience for economic resilience has not yet been explicitly addressed.

There are certain empirical economic studies that point towards the relevance of looking at the link between mental and economic resilience. It has been shown that under shock conditions, cultural hysteresis explains the different reaction of places to the same/similar economic shocks (Tubadji et al. 2019,2016). It has also been debated whether the psychological types are constant over time or they are a subject to change (Obschonka et al. 2013; Stuetzer et al. 2014). Clearly, this links to the question of cultural persistence versus cultural change (Baddeley et al. 1998; Guiso et al. 2016; Tubadji 2021), which is also still an unresolved question, subject to undergoing debates in philosophy of language and narratives economics (Tubadji 2020a, b, c, e; Sacco 2020). Moreover, Milani (2020) and Tubadji et al. (2020a) both agree that national public policy affects public mental health and risk perceptions within the country, as well as across its neighboring countries. The current study is, however, the first of its kind to look explicitly at the microeconomic mechanism of culture as a source of mental resilience of the individual and by extension of the general public.

The role of culture as a source of stability and psychological comfort with socio-economic aftermaths is well known from studies on social capital and organizational culture. Social capital helped the deprived regions of Italy to find the means through cooperatives to pull themselves out of the economic deprivation (Helliwell and Putnam 1995; Siisiainen 2003). Organizational culture and management culture in risk management are essential for the productivity and creative flourishing of economic organizations (Denison and Spreitzer 1991; Hofstede 1998). Yet, all these aggregate-level economic studies only assume the existence of an individual mechanism, linking psychological states and economic outcomes without aiming to empirically explain why the link exists.

Secondly, on the micro level, neuroscience self-management with the use of culture (as a tool for maintaining personal balance and achieving further development) is related to the study of cultural practices as a type of a meditation practice (Sudheesh and Joseph 2000; Koen 2008). Namely, there is specific evidence that playing violin is related to better neurological conditions (Zatorre 2005; Juslin 2009) and increased brain plasticity (Johansson 2006). Culture seems also to build neurological resilience against dementia (Cohen 2009). Dancing in cases of dementia (Palo-Bengtsson et al. 1998), and generally music engagement, improves cognitive decline (Innes et al. 2016).

Neurological conditions are also associated with the general immune system of the person (Davydov et al. 2010; Pariante 2016), which might be strongly relevant in health emergencies and pandemics such as COVID-19. Therefore, this study argues here further that cultural participation serves to increase (through mental health) the overall immunity of the person. Put differently, cultural consumption serves for building the ability of the entire health of a person to be more resilient under increased uncertainty.

Yet, at the background of the above literature, several questions emerge. How are cultural consumption and cultural resilience exactly related? Is culture only a private consumption matter or is it an efficient tool to be provided by policymakers as prevention for mental health decline with importance on aggregate level? In other words, is culture to be practiced as a private opium dosing for alleviating the pain once stress has occurred (see IFACCA 2020), or is culture a prevention mechanism that has to be in place persistently and before the negative shock strikes the individual and the socio-economic system of people (see Kagan 2014; Holmes et al. 2020)? Put differently, is culture to be treated as a luxury, or as an essential public good to be preserved under any budget constraints and fiscal polity cuts, since it provides a crucial security net for general public mental health prevention. The answers to all these questions converge into the need for evidence that a microeconomic mechanism of impact of culture on mental health resilience exists.

## A CBD micro model for culture and public mental health

Throsby (1999) has pointed to the cultural and economic valuation of assets, where the economic valuation accounts for the cost of the inputs, while the cultural valuation accounts for the perceived value added that the asset has to the socio-economic life of individuals and society. Based on this, CBD argues here that culture has been significantly under-evaluated in public policy and investment considerations on policy level, due to being evaluated only in its direct economic value, associated with generating profit. Meanwhile, culture has an indirect value—which divides into two parts. The one is the indirect economic impact of culture on other economic processes such as innovation, entrepreneurship, social entrepreneurship, and smartness of a city (Caragliu et al. 2011; Caragliu and Nijkamp 2011; Tubadji and Nijkamp 2016; Tubadji and Montalto 2020). The second indirect value of culture is a cultural valuation aspect, related to the impact of the cultural milieu (the attitudes) on the social aspects of the mental health of people. This second value of culture is the target of the modeling approach in this paper.

Ponticelli and Voth (2020) offer a study on macro level in this direction—showing that fiscal policy interventions, and not economic policy interventions (such as increase of taxes), are the measures associated with social unrest. Put differently, it is not only the economic cost that matters for the feelings of the public but also the cultural value associated with the social meaning of the policy measures. It is important for people whether the public interest or the private interest is benefitted by the policy measures. This affects the psychological reaction of the people (i.e., the electoral vote and the behavior of the masses) in response to policymaking. The link between fiscal policy and the feelings of left behind has been demonstrated also in the context of Brexit (see for instance Rodríguez-Pose 2018). There are even rare studies documenting the existence of this mechanism on individual level (Lee et al. 2018; Tubadji 2020b). Tubadji et al. (2020b) and (2020c) have looked at the link between cultural fiscal policy cuts, austerity, and ultra-right voting. All these studies demonstrate that one can expect a link between fiscal policy for the arts, mental health on aggregate level, and socio-economic aftermaths from overlooking this link. However, there seems to exist a gap in the literature with regard to empirical analysis on micro level data linking cultural participation (the result of supportive cultural policy for the arts) and individual mental health. The current study aims to address this gap by providing a hedonic micro model for testing that particular matter.

The CBD-inspired hedonic model, proposed in this study, has three main postulates. It starts with the CBD definition of cultural capital that distinguishes between living culture (current culture and art attitudes and assets) and cultural heritage (inherited attitudes and assets from the past) (Tubadji 2012; 2013). Our model builds on existing evidence that living culture and Bohemians are associated with creativity and mind plasticity, while cultural heritage is the more rigid component, linked to certainty-building feelings of identity, but associated with less creativity and less innovation (Tubadji and Montalto 2020). Based on this, CBD postulates that:

1. Living culture, consumed through cultural participation, is the source of mental health resilience;
2. Cultural heritage is a source of stability of one’s perception for identity, but needs to be in amounts lower than living culture, in order to allow for brain plasticity^6^; and
3. Cognitive bias towards under-valuation of culture in its indirect cultural and economic value for society leads to the oversight of culture as a tool for prevention of mental health during negative shocks to the economy.

The mechanism behind the above CBD postulates can be expressed as a microeconomic utility model that underlies the behavior of agents in the socio-economic system:

1 $$U=f\left(C,Y,D\right)$$

where *U* is the utility of the consumer, which can be defined as their life-satisfaction and mental health condition (assuming that happier people are in a better state of satisfaction with life and in a better mental health); *C* is the vector of cultural valuation of life, which stands for the need for culture, inspired by our love for certainty; this is strongly positively associated with cultural heritage and identity through the mechanism of love for homogeneity (i.e., like all mammals, people feel more secure when surrounded by our own herd and its cultural symbols (see Hall 1966)); it is also related to living culture, through the brain plasticity that cultural participation increases and generates potentials for resilience under stress conditions; *Y* is the vector of economic valuation of life, which includes the income of the person, occupation, their educational level, and labor market status; *D* is a vector of demographic characteristics, such as gender, age, marital status, having children.

Under negative socio-economic shocks—such as the COVID-19 pandemic—a cultural hysteresis is documented to exist in entrepreneurial response to the shock due to differences in cultural identity (Tubadji et al. 2016). For example, in the economic crisis 2007, Greek youth became less entrepreneurially inclined, while German youth became more so (Tubadji et al. 2019). The current paper argues that across individuals from the same cultural background, the response to the shock also differs due to their differences in mental health resilience. It is known that such differences exist among entrepreneurs (Hopenhayn and Vereshchagina 2003; Ucbasaran et al. 2009). Yet, it is assumed this variation is exogenous. Our study argues that mental resilience is (i) varying across the entire population (not just entrepreneurs), and (ii), this variation is not exogenous. It is endogenous, because the cultural participation is a tool through which the mental health resilience of the individuals can be and is intervened.^7^ Therefore, our main expectation is that cultural participation affects the *C* component of model (1).

Last but not least, if culture affects the mental health of individuals in the aspects that concern their willingness to and choice for cooperation with others, this clearly represents a strong evidence for the relevance of the micro-effects of culture for the macroeconomic environment. This is so, as according to Schelling (1969, 1978), discriminatory individual choices can have a profound and intensified effect on the segregation of society on the macro level. Segregation and discord in times of increased uncertainty is clearly an undesirable macro result.

## Happiness in COVID-19 times: And empirical operationalization of the CBD model

### Data

The data used for the main empirical tests in this study is based on a pilot survey, disseminated online in the beginning of the pandemic COVID-19 period, namely between 23 and 29 March 2020. The survey has five sections, requesting information on happiness and life-satisfaction, exposure to art and cultural consumption, exposure to human interaction, social capital and altruism, and an experiment with impact of art on happiness in COVID-19 times. The questionnaire contains also questions about demographics on individual and household level.

Our main outcome of interest is mental health resilience. It is approximated through a series of variables quantifying happiness, in its short- and long-term (life-satisfaction) dimensions, as well as propensity (i.e., happiness) to help others.

Short-term internal experience of happiness is measured through a question about the level of happiness self-reported on a Likert scale from 1 to 10, about happiness feelings experienced on the day of responding to the survey. The long-term form of happiness is measured according to three alternative concepts of long-term happiness—i.e., the three key concepts for life-satisfaction, flow, and meaning. These are based respectively on Kahneman and Krueger (2006); Frey and Stuetzer (2018); and Weimann et al. (2015). They can help to disentangle happiness before and during the pandemic. I have also an additional special control variable that may affect the report on happiness in the moment of response (as noted relevant by Levinson 2013)—namely a control for weather conditions.

The alternative outcome variable of interest which is used in this study stands for the happiness (or readiness and propensity) of the person to help other people. It captures the external behavioral aspects of happiness. I have data on propensity to help a stranger in the past and during the pandemic period. This allows me to measure the change in social capital propensity due to the loss of certainty under the COVID-19 pandemic shock, which reflects the resilience aspect of our measure.

The culture-related variables available in the dataset have three dimensions. First, I have information about the country of origin of the respondent (most responses coming from the UK, USA, or Japan). As these are countries that experienced serious blows from the pandemic, I consider the data relevant for the intended pilot study. Second, I have data on cultural participation—both public and private versions of it. I have participation in “publicly” provided free online access to cultural heritage (museum visits) and living culture experiences (concerts) (i.e., with no incurred economic cost, and therefore, supposedly the economic valuation does not differ and can be regarded as at a ceteris paribus condition). I have information on private experiences related to culture, such as singing with others (as the behavioral pattern was from Wuhan communities (BBC 2020), throughout Italy (Kearney 2020) and also compassionate citizens from neighboring countries (Xinhua 2020) singing to support each other’s moral during the lockdown). Third, attempting to quantify fully the CBD definition for culture (Tubadji 2012, 2013), the survey has collected information about past cultural consumption behavior related to frequency of visits to live cultural events.^8^ This data helps to distinguish between the effects of living culture and cultural heritage, i.e., the different components of culture, as well as the temporal difference in culture as an emergency tool for alleviation of pain from uncertainty or a tool for long-term prevention of mental health which breeds psychological resilience among the members of the society before the crisis strikes.

A set of socio-economic characteristics (such as age, gender, level of education, marital status, number of people in the household, number of children in the household, labor market status, and occupation) are available as control variables. All variables used in the analysis of this study are presented with definitions and descriptive statistics in Appendix Table 6. The full questionnaire is available as Appendix 3.

To capture the exogenous influence from the shock that COVID-19 represents, I use the cumulative number of deaths from COVID-19 on the day of response to the survey for each individual.^9^ This data is obtained from the COVID-19 dashboard of the World Health Organization.

Finally, there is available aggregate data from Google Trends regarding searches in Google about the validated positive psychology word “anxiety,” which stands for the mental health state of the searching individual, as well as the self-explained word “death.” This study uses this linguistic signifier of meaning and mental health on aggregate level and links it to indicators of socio-economic development (in this case public spending on cultural services). This follows the linguistic narrative economics of meaning CBD approach (see Tubadji 2020a, b, c, d, e for more details on this approach). This approach has been applied in its full extent elsewhere—addressing on the aggregate level the study of mental health and public policy during the pandemic periods across different countries (Tubadji et al. 2020a). Here, it is used only for descriptive comparison, to ensure some validation and generalizability check of the microeconomic results. It serves to compare the anxiety levels experienced in Germany, a country which both traditionally and now during the COVID-19 increasingly supports its art sector, as opposed to other EU countries, which gradually support the cultural sector less. While this macro-inference here relies on associations and should be subject to further analysis, it seems interestingly in line with our micro-findings by clearly illustrating which countries experienced higher anxiety during the same pandemic shock.

### Method

There are three main sub-types of cultural impact that need to be tested according to the above-stated CBD postulates. These three impacts relate to (i) the effect of the cultural consumption (living culture and cultural heritage) on happiness in COVID-19 times; (ii) the difference between past and present consumption of culture on happiness in COVID-19 times; and (iii) the difference between public offer consumption and private engagement in culture as a hobby and the effect of culture on happiness in pandemic times. Additionally, I would like to test the relationship between the impact of cultural consumption and the pro-social capital propensity of the individual during the pandemic period. These expected relationships can be stated as four main testable hypotheses, as follows:

- H01: Present cultural consumption impacts individual happiness during COVID-19 times.
- H02: Past cultural consumption impacts individual happiness during COVID-19 times.
- H03: Present cultural consumption impacts individual propensity to social capital during COVID-19 times.
- H04: Past cultural consumption impacts individual propensity to social capital during COVID-19 times.

Each of these hypotheses can be tested through an alternative operationalization of culture, as follows. To distinguish the public and private aspects of the experienced cultural participation and consumption, the public aspect will be operationalized through visit to art events (in pre-pandemic times) or online art consumption during the pandemic. The private aspect will be operationalized through personal engagement in art hobbies before or during the pandemic, as well as singing with others. These variables can be used separately, as determinants for the outcome of interest, and shall be ultimately horse-raced against each other in one multiple regression. The most parsimonious latter specification will be reported in the Results section.

In a first step, for testing hypotheses H01 and H02, I estimate a multiple regression, using OLS with robust standard errors. This means that I operationalize model (1) in the following manner:

2 $$Happiness\_during\_COVID-19=\alpha +{\beta }_{1}C1+{\beta }_{2}C2+{\beta }_{3}C3+{\beta }_{4}Y+{\beta }_{5}D+{e}_{1}$$

where self-reported *Happiness_during_COVID-19* times is *U* from model (1); the component *C* is quantified in a filigree manner to reflect: *C1*, the different types of cultural impact that I am interested in (namely, the type of event watched online—related to concert (living culture) or museum (cultural heritage); *C2*, the past engagement in cultural activity based on public offer such as concerts and theaters; *C3*, singing alone activity during COVID-19 times (which does not depend on any economic or public provision, but rather on the cultural valuation of the cultural experience by the individual); *Y* is alternatively quantified either by self-reported income or by degree of education, as these might be strongly correlated*. D* is a vector of our demographic control variables, including age,^10^ gender, marital status, information on whether the individual has children, and type of area one lives in (rural or urban).

Similarly, to test H03 and H04, I assume that propensity to social capital, altruism, and reciprocity can be regarded as utility, or happiness to help a stranger during COVID-19 times. This has been seriously analyzed in close relationship to resilience as well elsewhere (Trosper 2009; Zahran et al. 2011). Therefore, I use again model (1), operationalized this time as follows:

3 $$Social\_Capital\_COVID19=\alpha +{\beta }_{1}C1+{\beta }_{2}C2+{\beta }_{3}C3+{\beta }_{4}Y+{\beta }_{5}D+{e}_{2}$$

where *U* is quantified here as a propensity to help a stranger during COVID-19 times, as well the eventual decrease or increase of this propensity in comparison to the individual’s propensity to do so in the past. The explanatory variables are the same as in model (2).

I estimate model (2) using an OLS with country fixed effects to account for the cultural differences and state policy for handling the pandemic. To estimate model (3), I use an OLS when I employ the levels of the variable regarding social capital. When I estimate model (3) with dependent variable the decrease or increase of social capital in comparison to the “pre-pandemic” social capital propensity of the individual, I use a Probit model as these are binary outcomes. In order to account for the cultural heterogeneity across space, I use country dummies to account for the fixed effects in both OLS and Probit estimations, and across all our estimations discussed hereafter.

The first step of the analysis uses some additional control variables to capture (i) the eventual confounding influence of unemployment; (ii) the relationship of mental health resilience both with happiness and with life-satisfaction which can be compared in order to disentangle the primary psychological endogeneity concerns (discussed in detail in the “Results” section). In all estimations, I include also a control for the exogenous factor that COVID-19 represents. This is the cumulative number of deaths from COVID-19 on the day of the response to the survey.

In a second step, the study delves further into the economic endogeneity concerns about the cultural consumption. Following similar logics as Altonji et al. (2005), I consider the potential dependencies between (i) happiness and cultural consumption and (ii) income. To do so, I first cross-check the correlation between the happiness variables and income, education, and type of place of living (urban versus rural). I also cross-check whether the consumption of culture in normal times is associated with people’s preferences for art as a hobby. Furthermore, I disentangle the relationship between expectations for the end of the pandemic and the cultural consumption prior to the pandemic, as a robustness check whether mental health resilience (approximated through positive prospect to the future) and cultural consumption are statistically associated. Pearson pairwise correlation coefficients are considered with regard to all these additional variables and past cultural consumption.

In a third step, the paper explores the heterogeneity of the happiness reported in COVID-19 times. I cross-check whether there is similar heterogeneity of the life-satisfaction variable, when measured with our alternative three measures of happiness. In the presence of a heterogeneity, a sample selection model requires to be applied.

In a fourth step, the study explores (a) the preselection into being happy during COVID-times, based on the previous consumption of culture, and (b) the level of happiness during COVID-19 reported by the individual, including a correction term for the preselection in (a). To do so, I test models (2) and (3) using a Heckman sample selection model. In its first equation, I model preselection, explaining above average happiness as a function of past consumption of culture. I obtain a correction term from this estimation and use it as an additional regressor in model (2) and model (3), respectively. The second equation of the Heckman selection model is the augmented with this correction term model (2) or model (3), depending if I want to test H01 and H02 or H03 and H04, as described above.

Finally, in a fifth step, in order to appreciate more fully the potential link between the individual mechanism of culture as a tool for resilience and the aggregate effect of it for the entire population, the study employs some aggregate data for daily search of anxiety-related terms in Google and macro data on cultural and other types of public spending. A full-fledged empirical exploration with such data is available in Tubadji et al. (2020a). Here, the aggregate data is used only to put our micro results into perspective and appreciate their potential implications on macro level for further research.

### Results

#### Culture and happiness in COVID-19 times

Table 1 presents four specifications. Specification 1 represents estimation for happiness in COVID-19 times, specification 2 explains level of social capital propensity in COVID-19 times, specifications 3 and 4 check for the confounding effect of unemployment on the results, while specifications 5, 6, and 7 clarify the relationship of the results with general level of life-satisfaction. Specifications 8 and 9 explain respectively decrease and increase in the propensity to social capital in comparison to the pre-pandemic period. As the latter two specifications are estimated with a Probit model, marginal effects at means are presented.

**Table 1:** Happiness and social capital during COVID-19

Method	OLS—parsimonious		OLS—unemployed		OLS—happy_always			Probit	
	Happy during COVID-19 (in levels)	Helpful to others during COVID-19 (in levels)	Happy during COVID-19 (in levels)	Helpful to others during COVID-19 (in levels)	Happy during COVID-19 (in levels)		Helpful to others during COVID-19 (in levels)	Increased helpfulness to other	Decreased helpfulness to others
	Coef	Coef	Coef	Coef	Coef	Coef	Coef	dy/dx	dy/dx
Practiced art activity during COVID-19 lockdown	0.366	0.649	0.601	0.388	0.313	0.342	0.656	0.035	− 0.003
Attended online concert	0.006	− 0.472	− 0.515	0.026	− 0.096	− 0.104	− 0.459	0.050	− 0.020
Attended online museum	− 0.105	− 0.667	− 0.691	− 0.094	− 0.195	− 0.152	− 0.656	0.128	0.105
Age above 45 years	0.628	1.411	1.440	0.614	0.756	0.720	1.396	− 0.008	− 0.063
Female	− 0.240	− 0.207	− 0.288	− 0.202	− 0.328	− 0.332	− 0.196	− 0.012	0.226*
Lives in city	− 0.060	− 0.840	− 0.801	− 0.078	− 0.061	− 0.096	− 0.840	0.115	0.107
Highest education: PhD	0.620	− 0.051	0.021	0.586	0.030	0.069	0.020	0.045	0.080
Highest education: Master’s degree	0.452	− 0.380	− 0.272	0.402	− 0.217	− 0.149	− 0.300	0.023	− 0.036
Highest education: Bachelor’s degree	− 0.096	− 0.883	− 0.790	− 0.139	− 0.565	− 0.566	− 0.826	− 0.016	0.082
Married	0.400	− 0.355	− 0.339	0.393	− 0.097	− 0.199	− 0.295	− 0.014	− 0.075
With children	0.079	− 0.217	− 0.200	0.071	− 0.329	− 0.168	− 0.168	− 0.211	0.203
Sang with others during COVID-19 lockdown	0.460	0.418	0.412	0.463	0.193	0.203	0.451	0.127*	− 0.291**
Sunny on day of response to questionnaire	0.231	1.331*	1.356**	0.219	0.317	0.313	1.320**	− 0.102	− 0.077
Health-insured	− 0.206	− 0.808	− 0.730	− 0.243	− 0.150	− 0.096	− 0.815	− 0.075	0.053
Level of cultural consumption pre-pandemic	0.194***	0.093	0.105	0.189***	0.066	0.066	0.109	0.012	− 0.023
COVID-19 deaths (total number on day of response)	− 0.001	− 0.001	− 0.001	− 0.001	− 0.001	− 0.002*	− 0.001	0.000	0.000
Unemployed			0.390	− 0.182					
Life-satisfaction					0.632***	0.559***	− 0.076		
Interaction: life-satisfaction and COVID-19 deaths (total)						0.000			
USA	0.444	− 0.372	− 0.381	0.449	0.797	0.709	− 0.414	− 0.136	− 0.048
UK	1.174	− 0.020	− 0.009	1.169	1.247	1.196	− 0.029	0.106	− 0.071
Japan	1.360*	− 0.267	− 0.433	1.437**	1.304**	1.368**	− 0.260	− 0.054	− 0.088
Sweden	1.629*	− 1.655	− 1.654	1.629**	1.790***	1.795***	− 1.675	− 0.050	− 0.014
Spain	4.065	4.650	4.561	4.106	3.573	2.584	4.709	0.196	− 1.480
Italy	6.246	5.391	5.377	6.253	5.518	4.037	5.479	0.386	− 2.026
Albania	0.590	3.113	3.254*	0.524	0.639	0.712	3.107	0.043	− 0.384
Canada	− 0.766	− 2.066	− 2.090	− 0.755	0.470	0.389	− 2.215	omitted for coll	− 0.067
China	4.363	2.122	1.752	4.536	3.630	2.753	2.211	omitted for coll	omitted for coll
Germany	0.690	2.502*	2.548**	0.669	1.038	0.993	2.460*	0.469**	omitted for coll
Constant	4.396***	5.667	5.395***	4.523***	0.714	1.162	6.111***		
*N*	153	153	153	153	153	153	153	144	145
*R*-squared	0.28	0.22	0.22	0.28	0.48	0.49	0.22		

The table presents OLS estimates with robust standard errors and country of origin fixed effects. *p < 0.1; **p < 0.05; ***p < 0.01

As seen from Table 1, neither the economic-valuation-free art activities at home nor the economically free online living culture or cultural heritage activities are associated with the happiness of the individual in the pandemic period. However, the pre-pandemic consumption seems to exhibit a very strong positive association with the mental resilience of the person under shock conditions. When I look at the propensity to social capital, there is no effect on the levels in specification 2, because the pandemic increased the pro-social propensity of some people and decreased it with others. This differs across countries and across individuals.^11^ Specifications 3 and 4 show that our results seem not sensitive to unemployment level. According to specification 5 to 7, the general life-satisfaction seems to explain the happiness level during the pandemic, overriding the effect of cultural consumption. Yet, the interaction of life-satisfaction with the number of deaths is not significant, although number of deaths itself became a significant factor for happiness. Thus, it seems that life-satisfaction has a strong relationship with culture but is not the reason for mental health resilience during the pandemics. Also, life-satisfaction does not have any bearance on the propensity to help others. I interpret this as evidence that personality type (i.e., generally satisfied with life person) might be a very strong predictor of happiness. Yet, while the effect from it cannot easily be clearly distinguished from cultural consumption during the pandemic, it certainly does not affect the mental resilience and social capital propensity of the individual during this period. Past cultural consumption instead seems to matter for the resilience of mental health, since it takes away the effect from the number of deaths on happiness, in a “horse-race” empirical scenario setting between these two determinants. Moreover, when I look separately at the increase and decrease of pro-social propensity, specifications 8 and 9, respectively, I see that the spontaneous cultural expression of singing with others during the pandemics has a positive association with the pro-social behavior of the individual and relates to less often loss of pro-social propensity. This is a clear sign that engaging in a cultural practice during the negative shock is positively associated with the resilience of the social capital propensity during the pandemics. Meanwhile, women seem to be associated with higher loss of pro-social propensity due to the increased uncertainty during the pandemic. As further statistical checks demonstrated (results available upon request from the author), women have a higher propensity to help pre-pandemic, and therefore the corresponding loss due to the shock is higher for women during the pandemic. The exogenous factor—number of deaths—does not affect the results.^12^

In short, the cultural consumption pre-pandemic seems to increase the individual short-term level of happiness during the pandemic, while the cultural practice during the pandemic affects (more precisely, increases and even prevents loss of) the pro-social behavior during the pandemic. The strongest predictor of this mental health resilience seems to be the consumption of culture before the pandemic period, with a coefficient of impact on happiness amounting to 20%. It seems therefore that I cannot reject our H01 and H03, while the other two hypotheses do not find support in our findings. Meanwhile, it seems that both past cultural consumption and present cultural consumption have their associations with different aspects of the mental health reaction of the individual during the pandemic period. These findings point that culture could be both a tool for ensuring promotion of individual mental health and resilience of social capital.

#### Endogeneity of cultural consumption

In order to explore the potential economic dependence of our main explanatory variable (culture) and our outcome of interest (happiness) on income, I compare the strength of correlation among several variables in our models (1), (2), and (3). I engage in simple regressions and pairwise correlation explorations of the cultural consumption in pre-pandemic period and, respectively, income, education, life-satisfaction (in all the three aspects that I have discussed above), preference for art as a hobby, and expectations for the end of the pandemic. Tables 2, 3, and 4 present the results of these further explorations.

**Table 2:** Endogenous sources of happiness during COVID-19—correlations

	Level of cultural consumption pre-pandemic	Expected length of lockdown	Income	Highest education: PhD	Highest education: Master’s degree	Highest education: Bachelor’s degree	Female	Lives in city	Practices art as a hobby
Level of cultural consumption pre-pandemic	1								
Expected length of lockdown	− 0.16	1							
Income	0.04	0.06	1						
Highest education: PhD	0.05	− 0.07	0.32	1					
Highest education: Master’s degree	− 0.03	− 0.06	0.14	− 0.2361	1				
Highest education: Bachelor’s degree	0.01	0.11	− 0.07	− 0.3009	− 0.3342	1			
Female	− 0.03	− 0.04	− 0.20	− 0.18	0.13	0.11	1		
Lives in city	− 0.01	− 0.11	0.13	0.04	0.08	0.07	− 0.07	1	
Practices art as a hobby	0.15	0.02	− 0.11	− 0.11	0.06	0.00	0.01	− 0.09	1

The table presents Pearson pairwise correlation coefficients

**Table 3:** Endogenous sources of happiness during COVID-19—regression estimates

	Life-satisfaction (in levels)	Frequency of smiling (in levels)	Feels flow in work (in levels)
Age above 45 years	− 0.221	0.218	0.262
Female	0.153	0.264	− 0.060
Lives in city	− 0.012	0.113	0.012
Highest education: PhD	0.972*	0.194	0.974
Highest education: Master’s degree	1.069**	0.519	0.706
Highest education: Bachelor’s degree	0.767	0.280	0.864
Married	0.774	0.377	0.186
With children	0.658	0.082	0.622
Sang with others during COVID-19 lockdown	0.429	0.347	0.631
Sunny on day of response to questionnaire	− 0.101	0.111	− 0.406
Health-insured	− 0.113	0.158	− 0.359
Level of cultural consumption pre-pandemic	0.207***	0.232***	0.207***
COVID-19 deaths (total number on day of response)	0.000	− 0.002	0.000
USA	− 0.543	0.977	0.324
UK	− 0.115	1.174	0.708
Japan	0.075	0.037	0.722
Sweden	− 0.297	1.590*	0.434
Spain	0.774	5.426	2.584
Italy	1.217	8.221	3.704
Albania	− 0.088	− 0.268	1.144
Canada	− 1.998**	− 0.571	− 0.580
China	1.203	5.979	1.939
Germany	− 0.591	− 0.329	− 0.329
Constant	5.896***	5.286***	4.937***
*N*	153	153	153
*R*-squared	0.33	0.17	0.27

The table presents OLS estimates with robust standard errors and country of origin fixed effects. *p < 0.1; **p < 0.05; ***p < 0.01

**Table 4:** Endogenous sources of happiness during COVID-19—alternative sources of endogeneity

	Expected length of lockdown	Level of cultural consumption pre-pandemic					
Level of cultural consumption pre-pandemic	− 0.570**						
Income		0.044					0.050
Highest education: PhD			0.288				0.295
Highest education: Master’s degree							− 0.165
Highest education: Bachelor’s degree							0.120
Female				− 0.149			− 0.065
Lives in city					− 0.038		− 0.005
Practices art as a hobby						0.685*	0.743*
Constant	9.712	2.808	3.008	3.137	3.088	2.672	2.338
*N*	154	154	154	154	154	154	154
*R*-squared	0.027	0.002	0.002	0.001	0.000	0.021	0.029

The table presents OLS estimates with robust standard errors and country of origin fixed effects. *p < 0.1; **p < 0.05; ***p < 0.01

Table 2 presents the correlation coefficients. I see that past cultural consumption has a somewhat positive correlation with the hobbies of the person but with no other potential source of economic or demographic endogeneity such as income or gender. Yet, it has a clear highest correlation with the expectation for the length of the lock down period. This means that cultural consumption from pre-pandemic period can be expected to be a strong explanatory factor for the response of the individuals to the shock of the pandemic in terms of happiness and expectations for the future.

Demography and consumption factors may also be co-founded on one’s psychology. To address this, Table 3 presents the relationship of the demographic and behavioral characteristics from model (2) as explanatory factors for the general long-term happiness of the individual. The intention here is to cross-check whether the factors used for explaining happiness in the period of the pandemic are not associated with the determinants of the general state of happiness of the individual, rather than being predictive for the state during pandemics. The reasons why the cultural consumption during pandemics is excluded from these regressions is the logical causal direction. As the consumption during the pandemic is a behavior that follows temporally the general state of happiness of the people it cannot explain it. I find that cultural consumption from the pre-pandemic period is the only factor associated with all three measures for long-term individual happiness. This explains our results from Table 1 specifications 5 and 6, showing that long-term happiness is also a function of past cultural consumption, evidencing the cumulative effect of cultural consumption on mental health, claimed by the CBD model.

Table 4 shows that there is almost no other variable that significantly correlates with the past consumption of culture except the happiness of the individual and the present expectations for the end of the pandemic crisis. This is a strong indication for the exogeneity of the cultural consumption from past period with regard to economic influences. Therefore, culture seems to have acted as a plausible tool for mental health prevention in the group under investigation. Also, I see that the more the culture was consumed in the past, the shorter the expected lockdown period is. This result highlights the previously commented high correlation in Table 2a between cultural consumption and expectations. It suggests that an important association exists between the past cultural consumption and the expectations and mental resilience of an individual under shock conditions. This justifies trying to distinguish empirically between those people who had a higher and those who had a lower cultural consumption in the pre-pandemic period.

#### Heterogeneity of happiness in COVID-19 times

**Fig. 1:**
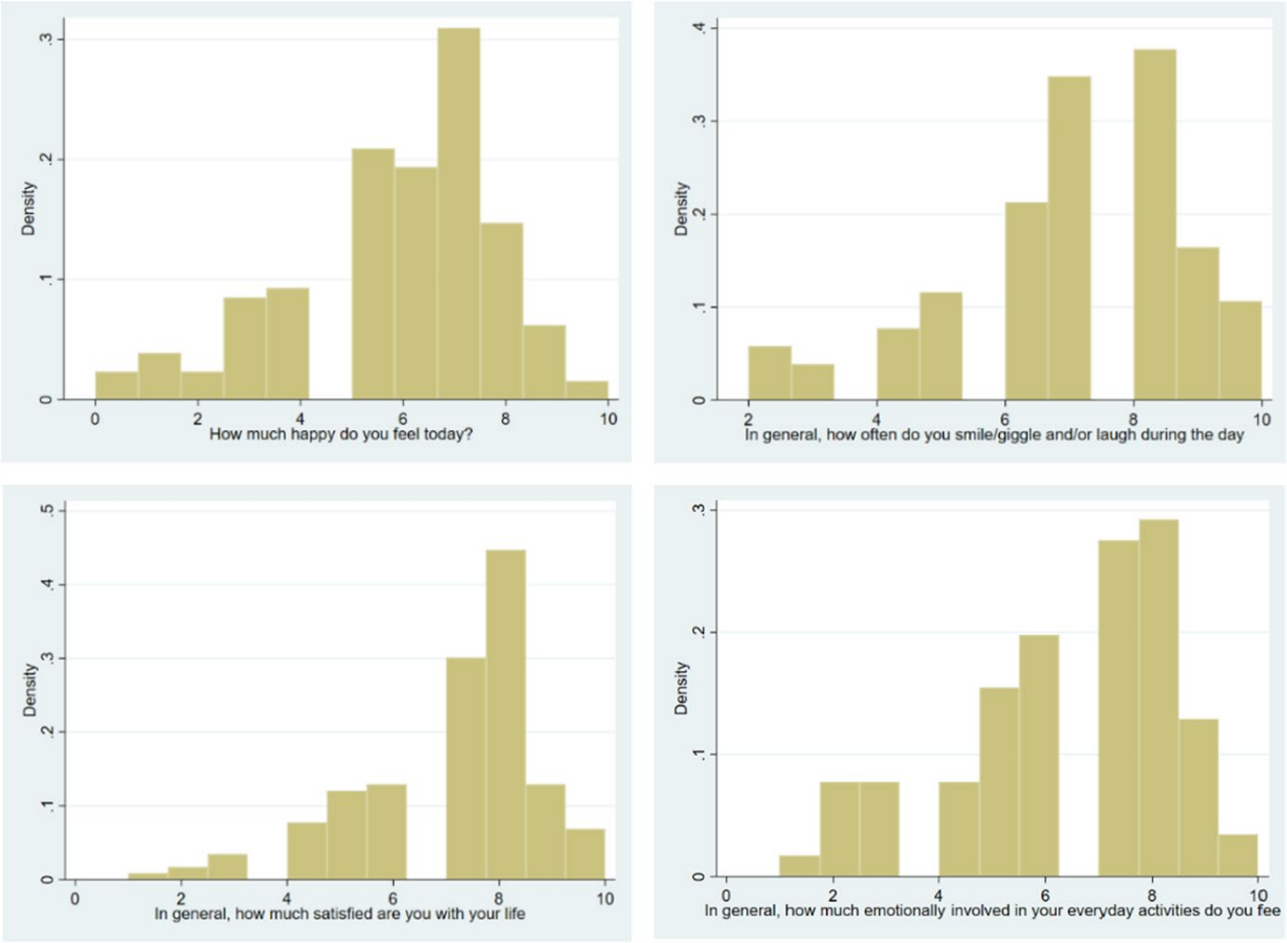
Distribution of daily happiness during COVID-19. Notes: The histogram presents the density of the response to 4 alternative happiness-related questions, measured on a Likert scale from 1 to 10. The questions are, clockwise from top left: (1) “How happy do you feel today?”, (2) “In general, how often do you smile?”, (3) “In general, how satisfied are you with your life?”, and (4) “In general, how much emotionally involved in your everyday activities do you feel?”

Using histograms to explore the density of the statistical behavior of our happiness and life-satisfaction data allows to delve deeper into the process under analysis. Namely, Fig. 1 shows the density of life-satisfaction (quantified through our three different measures) and happiness during the COVID-19 period.

As seen in Fig. 1, there is a clear presence of heterogeneity in our outcome variables of interest, namely a group of low and a group of high happiness. This pattern seems to be present with the life-satisfaction measures, although the division is most clear for the happiness during the pandemic period. Finally, I have learned from the preceding section that past consumption of culture is strongly related to the happiness levels. Past consumption of culture is associated also with preferences for cultural consumption, but not with any other potential factor for cultural consumption, such as income or education as previously discussed. Therefore, I have the statistical justification to question whether the happiness in COVID-19 is a subject of a sample selection bias driven by the past consumption of culture.

#### Cultural preselection for happiness in COVID-19 times

Three specifications of model (1) are estimated here through the use of a Heckman sample selection model. All specifications address the sample selection based on the past consumption of culture but differ in their dependent variable of interest. Specification 1 has as a dependent variable the level of happiness during the COVID-19 period, and specifications 2 and 3 have as a dependent variable the propensity to help a stranger (i.e., a proxy for social capital) as a dependent variable. The preselection is respectively done in specification 1 vis-a-vis being above average of the mean of happiness during the COVID-19 period; in specification 2, the preselection regards having your propensity to help others decreasing and in specification 3 having your propensity to help others during COVID-19 increasing in comparison to the usual such propensity in pre-pandemic times. The results are presented in Table 5.

**Table 5:** Cultural preselection for happiness during COVID-19

	Happy during COVID-19 (in levels)		Helpful to others during COVID-19 (in levels)		Helpful to others during COVID-19 (in levels)	
	Coef	dy/dx	Coef	dy/dx	Coef	dy/dx
Practiced art activity during COVID-19 lockdown	0.306	0.306	0.168	0.049	2.870***	2.870***
Attended online concert	− 0.079	− 0.079	− 0.038	− 0.011	− 0.835	− 0.835
Attended online museum	0.043	0.043	− 1.523	− 0.442	− 0.835	− 0.835
Age above 45 years	0.463	0.463	0.643	0.186	− 5.147**	− 5.147**
Female	− 0.292	− 0.292	− 0.740	− 0.215	− 2.186**	− 2.186**
Lives in city	0.120	0.120	− 1.339	− 0.388	− 2.340	− 2.340
Highest education: PhD	0.372	0.372	1.859	0.539	0.806	0.806
Married	0.085	0.085	− 2.010	− 0.583	3.940***	3.940***
With children	− 0.104	− 0.104	1.234	0.358	− 0.492	− 0.492
Sang with others during COVID-19 lockdown	0.280	0.280	− 0.075	− 0.022	− 5.509***	− 5.509***
Sunny on day of response to questionnaire	0.079	0.079	1.196	0.347	2.932**	2.932**
Health-insured	0.153	0.153	0.122	0.035	6.008***	6.008***
COVID-19 deaths (total number on day of response)	0.000	0.000	0.002	0.001	0.004	0.004
USA	0.310	0.310	1.347	0.391	− 1.178	− 1.178
UK	0.994*	0.994*	1.875	0.544	0.987	0.987
Japan	0.505	0.505	0.749	0.217	− 1.455	− 1.455
Sweden	0.551	0.551	0.964	0.279	3.194	3.194
Spain	1.678	1.678	− 3.883	− 1.126	− 7.765	− 7.765
Italy	1.479	1.479	− 7.552	− 2.190	− 10.146	− 10.146
Albania	0.811	0.811	2.308	0.669	4.422**	4.422**
Canada	*Omitted*	*Omitted*	2.770	0.803	*Omitted*	*Omitted*
China	1.376	1.376	− 3.165	− 0.918	*Omitted*	*Omitted*
Germany	0.101	0.101	*Omitted*	*Omitted*	0.617	0.617
Constant	7.047***		− 3.844		2.361	
Sample selection for	High happiness during COVID-19 (above mean level)		Decreased helpfulness to others		Increased helpfulness to others	
Level of cultural consumption pre-pandemic	0.096*		− 0.070		0.094*	
_cons	− 0.027		0.129		− 1.290	
/mills						
Lambda	− 1.233		6.771		0.457	
Rho	− 1		1		0.52	
Sigma	1.233		6.771		0.882	
* N*	153		153		153	

The table presents a Heckman selection model, where respondents to the survey are self-selected into higher happiness during COVID-19 times according to their preference to consume culture more often during non-pandemic times. Education is considered here only with a simple dummy distinction as otherwise there remains not enough statistical information to run the test. *p < 0.1; **p < 0.05; ***p < 0.01

Table 5 shows that indeed mental health (quantified either as individual happiness during pandemic times or the pro-social happiness to help others (propensity towards pro-social behavior)) is clearly associated with a preselection based on the pre-pandemic consumption of publicly provided cultural goods and services. That does not apply for the decrease of social capital, which is not associated in a statistically significant manner with the cultural consumption from the past. Yet, the sign of impact of past cultural consumption on the decrease of social capital seems to be indicating negative preselection for decrease of social capital. This is consistent with the fact that I find positive preselection effect from the cultural consumption for happiness and increase of social capital.

In terms of the corrected for preselection regressions, I see that our model explains best the increase of pro-social behavior during the pandemic. The increase seems positively associated with singing with others during the lockdown and the decrease is clearly negatively (though not significantly) associated with this variable. Meanwhile, our results for gender from the Probit model are confirmed here. Women are found less likely to venture into pro-social risky behavior during the pandemic. Interestingly, insurance becomes an important positive predictor for helping others during the pandemic period. Even more importantly, while I saw that singing with others increased the change towards pro-social behavior and decreased the likelihood to decrease pro-social behavior, when I take the preselection by past cultural consumption into account, it seems that the people who sang with others were less likely to help others per se during the pandemic period. This clearly indicates that cultural consumption in the past is associated with a boost of the pro-social behavior of those less likely to help others during uncertainty. The preselection also allows for the practicing of a cultural activity during COVID-19 to show its positive effect.

These results suggest that past consumption of culture can act as a shield for the individual mental health (expressed in higher levels of happiness for those having been on a higher cultural consumption level before the pandemic burst out). Moreover, cultural consumption seems not only associated with preservation but also with a significant enhancement of the mental resilience and propensity to help others; i.e., culture seems able to act as a potential tool for boosting of social capital during times of negative external shocks, such as the COVID-19 pandemic.

#### Reflections on implications for national cultural policy

In a final step, to help generalize and put into perspective the findings from the above explored CBD microeconomic mechanism, I find illustrated below the aggregate relationship between the cultural spending done over the period 2001–2018 and the mental health resilience of the countries on the aggregate level. This can be done by looking at respectively the governmental expenditure on cultural service for the period 2001–2018 (in Euros and in % of total national GDP) and comparing this with the intensity of using the search word “death” during the COVID-19 period in terms of mental health distortion through increased anxiety.^13^

**Fig. 2:**
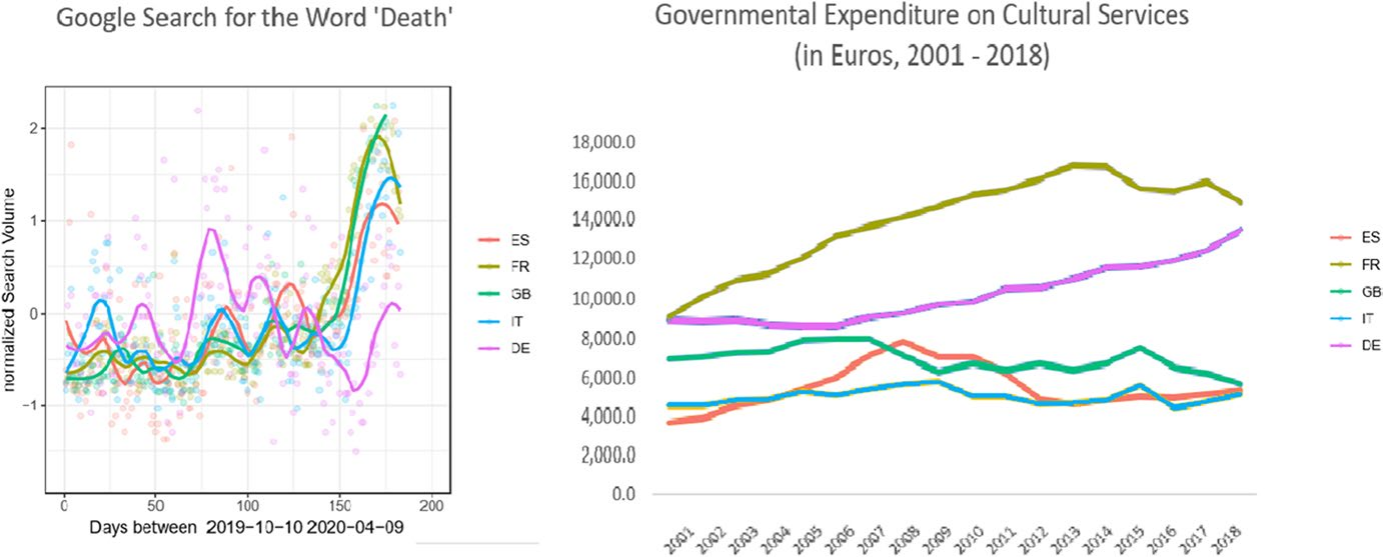
Anxiety from fear of death and country cultural policy. Notes: The figure uses Google Trends data about search for the word “death” during the COVID-19 pandemic period 01/01/2020–09/04/2020. Data on cultural policy spending is obtained from Eurostat

As seen from Fig. 2, for the six countries, Italy, Germany, Spain, France, and the UK (the countries with some of the biggest cultural sectors in the EU), Germany is the only country that increased its governmental spending both in terms of actual amount in Euros and in terms of percentage during the period 2001–2018 (for the latter, see Appendix Table 6). All other countries either decreased the spending or are at a lower level than Germany in real numbers spent on the cultural sector. Also, while Germany had an increase of other types of public spending as well (on education and health for example), the percentage increase of spending was highest in the case of culture (see Appendix Table 6).

Again, as visible from Fig. 2, Germany seems to be the country with the highest mental health resilience in response to the pandemic. During the period 1 January to 9 April 2020, this is the country where the increased search for the word “death” is the lowest.

Clearly, this is only descriptive aggregate-level illustration of the tendencies. Yet, there is evidence on the effect of public policy per se on mental health during the pandemic, which takes into consideration the number of deaths and other COVID-19-related state measures following the methodology of Tubadji et al. (2020a) and related studies such as that of Armbruster and Klotzbücher (2020). Meanwhile, the above figures demonstrate that there really exists some, at least anecdotal for the moment, evidence in the data that the here-explored micromechanism (of impact of culture on mental health of an individual during the pandemic period) seems to have a potential association on aggregate level with public mental health and public cultural spending as well. Thus, New Zealand might as well be a pioneer in public policy in pandemics also with regard to how it handles the cultural sector. Cutting-edge time series approaches developed for policy monitoring purposes during COVID-19 (see Bonacini et al. 2020a) can be applied for these available mental health– and culture-related time series to inform policymakers with higher precision.

## Conclusion

The current study examines the impact of the consumption of culture (both before and during the pandemic) on individual mental health and the resilience of the pro-social propensity in human behavior during the pandemic. The presence of such cultural impacts will demonstrate that culture is fundamental for maintaining a healthy social climate. The study explores the micromechanisms of cultural impact for both publicly organized and privately curated individual art engagement. It also compares the role of living culture and cultural heritage. The estimations rely on an OLS with fixed effects for country, a Probit, and a Heckman sample selection model.

The findings appear to support the hypothesis that past cultural consumption affects happiness levels during the pandemic. Additionally, during pandemics, art engagement seems to enhance pro-social behavior. The study first delves into the direct associations behind culture and mental health. Next, a detailed exploration of pairwise relationships clarifies concerns about the endogeneity versus exogeneity of cultural consumption. In addition, happiness levels show heterogeneity within the sample. This also appears to be strongly associated with individual cultural consumption before the pandemic period. Therefore, a Heckman sample selection model is estimated, where the selection into being happy is based on past cultural consumption. The study provides evidence for a preselection, namely, people have experienced greater happiness during the pandemic based on higher levels of consumption of culture in the pre-COVID-19 period. This preselection based on past cultural consumption cannot be rejected when the outcome variable of the model is the increase in the propensity towards pro-social behavior. Finally, the paper places the results of the study into a wider perspective by offering an illustrative snapshot of data on the aggregate level regarding the relationship between cultural services provision and the mental resilience of the general public during the pandemic in several countries.

The economic meaning of these results is that past consumption of culture during ordinary times might serve to create a mental health immune system, ensuring higher levels of mental health and happiness during negative external shocks, such as the COVID-19 pandemic. Meanwhile, present cultural engagement seems to be a potential way to foster the mental health of people during crisis periods and especially to enhance pro-social behavior during such challenging times. The causal direction between past consumption and general life-satisfaction merits further analysis.

These findings demonstrate that culture is associated with both individual- and community-related mental health through microeconomic behavior. As I know from agent-based modeling by Schelling (1969, 1978), a small change in microbehavior can account for a major change in the entire socio-economic system. Thus, by identifying a clear association between culture, mental health, and pro-social behavior on the micro level under uncertainty, this study suggests the potentially high significance of the cultural sector as a determinant of the aggregate psychological milieu on the macro level (i.e., for the general public’s mental health). On the aggregate level, it is widely acknowledged that culture is a significant factor for the socio-economic development of a place (as well known from Heilbrun 1992; Guiso, Sapienza and Zingales 2006, Tabellini 2010, Ottaviano and Peri 2006; Reggiani and Nijkamp 2009; Nijkamp and Reggiani 2012; Alesina et al. 2013; Tubadji and Nijkamp 2015; Tubadji et al. 2016; Alessina et al. 2013; Alesina and Giuliano 2015; Sacco 2020; Tubadji 2012, 2013, 2020a, b, c, d, e; Shiller 2017, 2019). However, to cross-check the validity of its effect on mental health at the aggregate level specifically, aggregate data are consulted here only illustratively to show the difference in mental health anxiety experienced in different countries. The link between the micromechanism analyzed in this study and the seemingly consistent tendencies in the macro level data merits further analysis.

The broader policy implications of the results of this study suggest that policymakers could use nudging techniques (which are already used in policymaking for supporting and promoting health prevention practices) to encourage people to consume more culture and to engage with cultural practices. The Banner of Peace Initiative, organized and led by Ludmila Zhivkova, Minister of Culture in Bulgaria, with the support of UNESCO, is an example of this type of cultural policy. This initiative was dedicated to nudging children around the world to engage with art for the promotion of international peace. The current study suggests that this might have been a good practice, given the findings about pro-social behavior and culture. The more prone people are to cooperate and value social capital in shock periods, the more prone they are to maintain peace. This topic is another potential extension of the current study with long-term implications.

Following the methodology of the pilot survey here, the next stages of the survey are intended to take place close to the end of the lockdown period, after the lockdown has been lifted, and 6 months after the end of the lockdown period. Better statistical power and causal analysis will potentially be possible based on these further data collection efforts. Additional analysis of the CBD microeconomic mechanism with regard to leisure time availability or job vulnerability can also shed more light on how this mechanism operates.

In conclusion, this study’s main contribution to the literature is the elucidation of a microeconomic mechanism, through which regular cultural participation behavior creates a mental health shield from uncertainty in shock periods and increases pro-cooperative behavior during crises. Multiple extensions of this micro level analysis and related macro level exploration represent novel pathways for further research.

## Figures and Tables

**Fig. 3:**
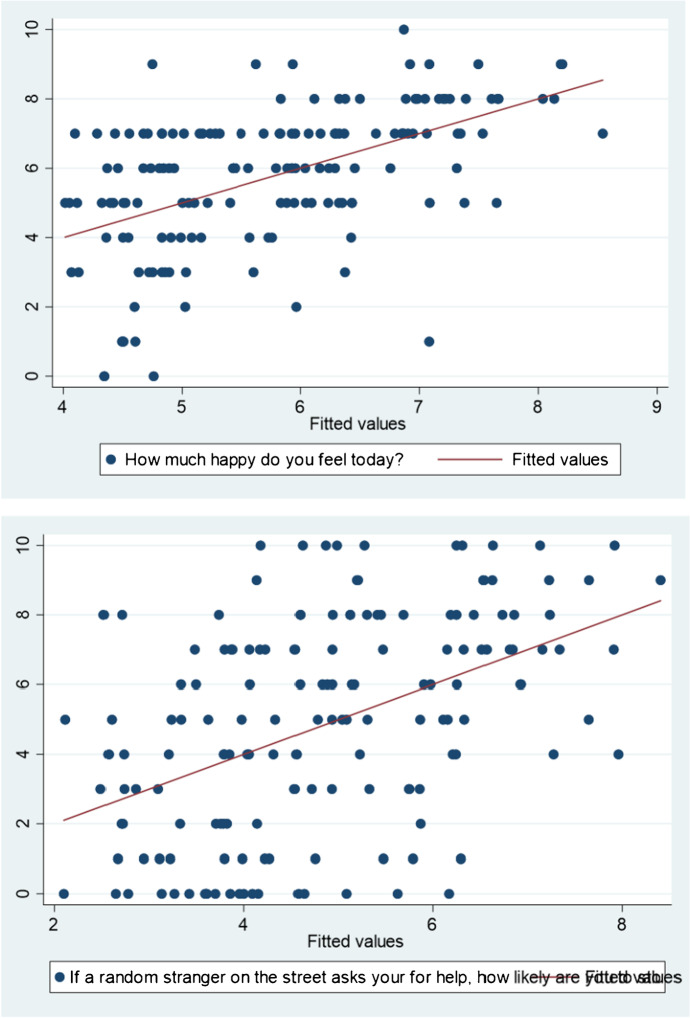
Main dependent variables and their predicted values. Notes: The figure presents the relationship between explanatory variable and its predicted value from an OLS regression with country fixed effects. The upper image presents this relationship for the level of happiness during COVID-19 times (i.e., variable “happy during COVID-19”) and its predicted value from the regression corresponding to the first specification in Table 1. The lower figure presents this relationship for the level of propensity to help a stranger during COVID-19 times (i.e., variable “help to others during COVID-19”) and its predicted value from the regression corresponds to the second specification in Table 1

**Fig. 4:**
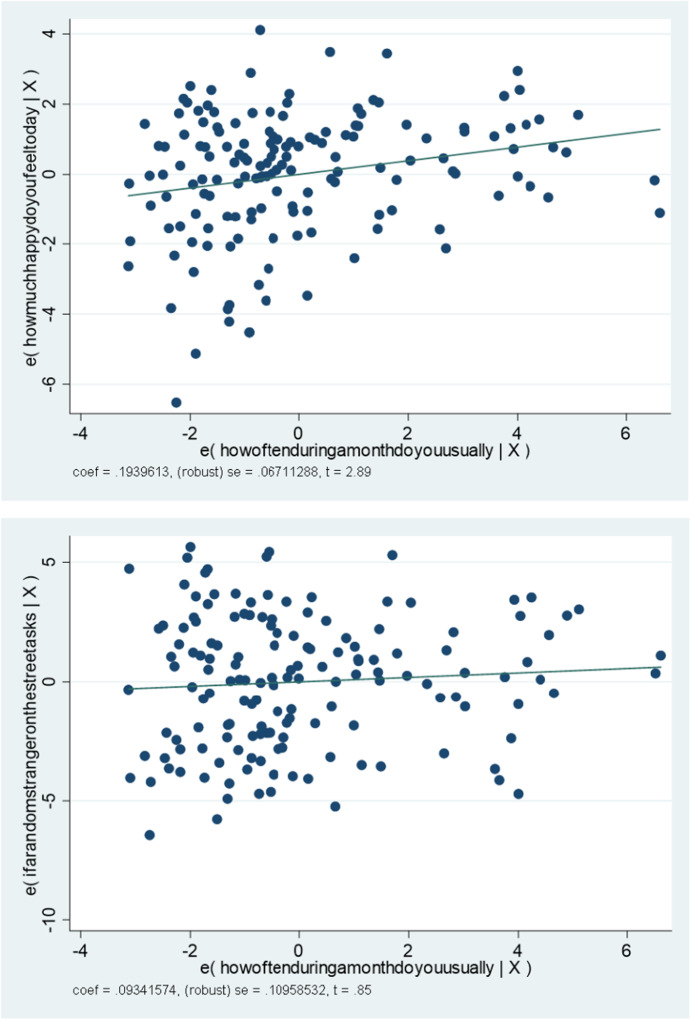
Main dependent variables and their dependence on past cultural consumption. Notes: The figure presents the added value plots, showing the relationship of all the explanatory variable in the OLS regression from Table 1 (specifications 1 and 2) and past cultural consumption

**Fig. 5:**
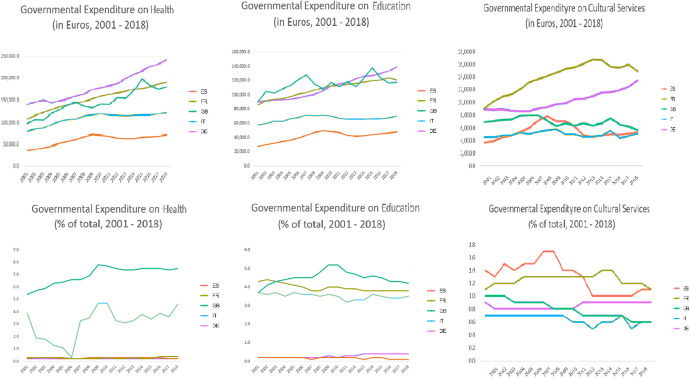
Expenditures on health, education, and culture. Notes: The figure presents the governmental expenditure in Germany, Spain, Italy, France, and the UK (i.e., the biggest European economies). It shows from left to right the expenditure on health, education, and culture. The top row represents the respective expenditure in millions of Euros. The second row represents the same expenditure as percentage of the total expenditure. The period covered by the data is 2001–2018. Source: Eurostat

## Appendix

**Table 6:** Descriptive statistics of main variables

Model component	Variable	Motivation	Obs	Mean	Std. dev	Min	Max
Happiness	Happy during COVID-19 (in levels)	How much happy the respondent feels on the today of survey	153	5.8	2.1	0	10
	Life-satisfaction (in levels)	Life-satisfaction (how much satisfied are you with your life)	153	7.0	1.8	1	10
	Frequency of smiling (in levels)	Savoring life attitude of happiness (how often smiling)	153	6.9	1.9	2	10
	Feels flow in work (in levels)	Flow as a measure of happiness (emotion in daily activity)	153	6.3	2.1	1	10
	High happiness during COVID-19 (above mean level)	Dummy equal to above mean of happiness on day of survey	153	0.6	0.5	0	1
Social capital	Helpful to relatives during COVID-19	Propensity to help a relative during COVID-19	153	7.2	3.1	0	10
	Helpful to a friend during COVID-19	Propensity to help a friend during COVID-20	153	5.7	3.1	0	10
	Helpful to a remote acquaintance during COVID-19	Propensity to help a remote acquaintance during COVID-21	153	4.8	3.1	0	10
	Helpful to strangers during COVID-19	Propensity to help a stranger during COVID-22	153	6.3	3.0	0	10
	Helpful to others pre-pandemic (in levels)	Propensity to help others measured on a Likert scale 0–10	153	6.2	3.0	0	10
	Helpful to others during COVID-19 (in levels)	Past propensity to help others measured on a Likert scale 0–10	153	4.9	3.1	0	10
	Change in helpfulness to others during COVID-19	Difference between the past and current propensity to help	153	− 1.4	3.4	− 10	9
	Decreased helpfulness to others	Dummy variable equal to 1 if propensity to help decreased	153	0.5	0.5	0	1
	Increased helpfulness to others	Dummy variable equal to 1 if propensity to help increased	153	0.2	0.4	0	1
Expectations	Expected length of lockdown	Expected length of lockdown in number of weeks	153	8.0	8.2	0	72
	Toilet paper amount envisaged as necessary	Number of toilet paper rolls needed	148	3.4	4.6	0	48
Culture	Level of cultural consumption pre-pandemic (living culture)	Frequency of visit to living culture event before the pandemic	153	3.0	2.3	1	10
	Level of heritage consumption pre-pandemic (heritage)	Frequency of visit to cultural heritage before the pandemic	153	2.3	1.9	1	10
	Attended online concert	Viewing online concert during COVID-19	153	0.2	0.4	0	1
	Attended online museum	Viewing online museum during COVID-19	153	0.1	0.3	0	1
	Practiced art activity during COVID-19 lockdown	Engaging with art activity at home during lockdown	153	0.5	0.5	0	1
	Sung alone during COVID-19 lockdown	Singing during pandemics period (alone) (dummy variable)	153	0.5	0.5	0	1
	Sung with others during COVID-19 lockdown	Singing during pandemics period (with others) (dummy variable)	153	0.3	0.4	0	1
Demographics	Income	Income on a Likert 0–10 scale, if average is 4	153	5.7	2.1	1	10
	Unemployed	Unemployed/without a job at the current moment	153	0.4	0.5	0	1
	Highest education: PhD	Dummy variable equal to 1 for having a PhD as last edu. level	153	0.2	0.4	0	1
	Highest education: Master’s degree	Dummy variable equal to 1 for having a Master’s degree as last edu. level	153	0.2	0.4	0	1
	Highest education: Bachelor’s degree	Dummy variable equal to 1 for having a Bachelor’s degree as last edu. level	153	0.3	0.5	0	1
	Married	Dummy variable equal to 1 if married	153	0.2	0.4	0	1
	With children	Dummy variable equal to 1 if having children	153	0.2	0.4	0	1
	Age	Actual age reported by respondent	153	28.8	8.9	18	55
	Age above 45 years	Dummy = 1 if above 45 years of age	153	0.2	0.4	0	1
	Female	Gender dummy	153	0.5	0.5	0	1
Other controls	Lives in city	Living in a metropolitan area	153	0.8	0.4	0	1
	Hobby art	Having a hobby related to the arts and culture	153	0.6	0.5	0	1
	Health-insured	Having a health insurance	153	0.8	0.4	0	1
	Sunny on day of response to questionnaire	Responding to questionnaire on a sunny day	153	0.5	0.5	0	1
Exogenous factor	COVID-19 deaths (total number on day of response)	Cumulative number of COVID-19 deaths on the day of filling in the questionnaire, obtained from World Health Organization dashboard data on COVID-19 by country	153	734.8	1256.0	0	6077
Geography	Albania	Country dummy to serve as fixed effect	153	0.0	0.2	0	1
	Canada	Country dummy to serve as fixed effect	153	0.0	0.2	0	1
	China	Country dummy to serve as fixed effect	153	0.0	0.2	0	1
	Germany	Country dummy to serve as fixed effect	153	0.0	0.2	0	1
	Italy	Country dummy to serve as fixed effect	153	0.0	0.2	0	1
	Japan	Country dummy to serve as fixed effect	153	0.1	0.3	0	1
	Spain	Country dummy to serve as fixed effect	153	0.0	0.2	0	1
	Sweden	Country dummy to serve as fixed effect	153	0.0	0.2	0	1
	UK	Country dummy to serve as fixed effect	153	0.1	0.3	0	1
	USA	Country dummy to serve as fixed effect	153	0.4	0.5	0	1

The table presents the main descriptive statistics for the variables from the World Values Survey used at the individual level in this study

Figure 3

Figure 4

Figure 5

## Footnotes

1 CBD suggests that abiding by a particular culture calms an individual’s fear of uncertainty, makes individual behavior more predictable for others, and offers the feeling of security of a safety net since it ensures the presence of a group that commits (based on shared cultural identity) to help the individual in times of adversity as a pay-back for her willingness to commit to the cultural rules, since thus her behavior becomes best predictable (and least uncertain) to the rest of the community.

2 See, for instance, Sable and Kling (2001) and Mazzanti (2002) for a further overview of the literature on culture as a mixed good.

3 See Guiso et al. 2006; Benabou and Tirole (2011); and Alesina and Giuliano (2015) for extensive literature reviews on empirical evidence about the impact of culture on the economic system. On the individual level, cultural capital endowment is responsible for the dynamics of socioeconomic mobility of people and their success in transforming their abilities into skills and human capital (Bourdieu 1986, 1973; Bourdieu and Passeron 1979; Tubadji et al. 2017). On the aggregate level, the cultural endowment of cities has been demonstrated to make a major contribution both through living culture (concerts, festivals, exhibitions, see Snowball 2007) and through cultural heritage memory and tourism aspects (see McKercher et al. 2005; Cerisola 2019). The creative class concept has also highlighted the link between regional economic development and city smartness, on the one hand, and the concentration of the cultural sector in a place, on the other hand (Florida 2002, 2005; Moeller and Tubadji 2009; Tubadji and Montalto 2020; Florida et al. 2017).

4 A potential reason for this is the traditional attitude towards the cultural sector as a generally sluggish economic growth sector. Due to this condition, termed Baumol’s disease (see Baumol and Bowen 1966; Cowen 1996; Baumol and Towse 1997; Heilbrun 2003; Last and Wetzel 2011), culture has always been treated as a luxury in times of economic downturn, with libraries, theaters, and other cultural venues being among the first to experience the effects of austerity measures (Bramall 2012; Kloosterman 2014; Newsinger 2015; Bracci et al. 2015).

5 There is literature on inequality in cultural participation (see for most recent contribution Mak et al. 2020). But this question is generally viewed as a niche boutique question of a luxury industry, rather than being considered as a major type of inequality with significant socio-economic implications. This may add to the inequalities that COVID-19 acerbates, as pointed by Bonacini et al. (2020b), Tubadji et al. (2020a, b, c, d, e, f) and elsewhere.

6 This CBD postulate is very closely related and building on the work on cultural heritage and social change effects stemming from the important contributions by Chang (2014).

7 In essence, this is the other side of the coin of the Marxist argument that culture can be used as a tool for power over the masses. However, this study adopts a more behavior economic and nudge policymaking implications perspective.

8 The questionnaire of the pilot survey used in this analysis contains also a question about past cultural heritage–related consumption. Yet, there was too little variation in the responses to this question to allow using the variable in the analysis.

9 While according to Depalo (2020) and others, death rates might not have been in reality that high, the salient numbers of deaths discussed in the media and related public policy measures might have significantly affected the perceptions, mental health, and behavior of people, as pointed among others by Qiu et al. (2020); Milani (2020); and Tubadji et al. (2020).

10 Following Blanchflower (2020), I use also age above 45, in order to capture the potential non-linearities. Different cut-off ages were used, but the results do not differ.

11 A Probit model was estimated also for no change in the propensity to help. This outcome is negatively associated with online museum during the pandemic, which can be interpreted as evidence for the power of culture to induce change in pro-social behavior.

12 Additional checks for the reliability of the estimations in Table 1 show that the results are not driven by outliers. See Appendix Table 6.

13 The author thanks Frederic Boy, Swansea University, for providing the linguistic mental health data on aggregate level.
